# Marker assisted selection of new high oleic and low linolenic winter oilseed rape (*Brassica napus* L.) inbred lines revealing good agricultural value

**DOI:** 10.1371/journal.pone.0233959

**Published:** 2020-06-04

**Authors:** Stanisław Spasibionek, Katarzyna Mikołajczyk, Hanna Ćwiek–Kupczyńska, Teresa Piętka, Krystyna Krótka, Marcin Matuszczak, Joanna Nowakowska, Krzysztof Michalski, Iwona Bartkowiak-Broda

**Affiliations:** 1 Department of Genetics and Breeding of Oilseed Crops, Plant Breeding and Acclimatization Institute-National Research Institute (PBAI-NRI), Poznan, Greater Poland, Poland; 2 Institute of Plant Genetics, Polish Academy of Sciences, Poznan, Greater Poland, Poland; Huazhong University of Science and Technology, CHINA

## Abstract

Development of oilseed rape (*Brassica napus* L.) breeding lines producing oil characterized by high oleic and low linolenic acid content is an important goal of rapeseed breeding programs worldwide. Such kind of oil is ideal for deep frying and can also be used as a raw material for biodiesel production. By performing chemical mutagenesis using ethyl methanesulfonate, we obtained mutant winter rapeseed breeding lines that can produce oil with a high content of oleic acid (C18:1, more than 75%) and a low content of linolenic acid (C18:3, less than 3%). However, the mutant lines revealed low agricultural value as they were characterized by low seed yield, low wintering, and high content of glucosinolates in seed meal. The aim of this work was to improve the mutant lines and develop high-oleic and low-linolenic recombinants exhibiting both good oil quality and high agronomic value. The plant materials used in this study included high-oleic and low-linolenic mutant breeding lines and high-yielding domestic canola-type breeding lines of good agricultural value with high oleic acid content and extremely low glucosinolates content. Field trials were conducted in four environments, in a randomized complete block design. Phenotyping was performed for wintering, yield of seed and oil, and seed quality traits. Genotype × environment interaction was investigated with respect to the content of C18:1 and C18:3 acids in seed oil. Genotyping was done for the selection of homozygous high oleic and low linolenic lines using allele-specific CAPS markers and SNaPshot assay, respectively. Finally, new high oleic and low linolenic winter rapeseed recombinant lines were obtained for use as a starting material for the development of new varieties that may be of high value on the oil crop market.

## Introduction

Oilseed rape, OSR, (*Brassica napus* L. var. *oleifera*, ssp. *napus*) also called canola [1] is one of the most important oil crops contributing a huge volume to the global production of oil (68 million metric tons (MMT) in 2017) next to soybeans (330 MMT) and followed by oil palm (64 MMT) (http://www.fao.org/faostat/en/#home). Rapeseed oil has been used for many years mainly for human nutrition and major improvements have been made for over 40 years through intensive breeding for enhancing the quality of seed and oil [2–4]. Canola oil is beneficial to human health and nutrition; it includes a very low amount of saturated fatty acids, palmitic C16:0 and stearic C18:0 (in total about 7%), and mainly mono- and polyunsaturated fatty acids, oleic C18:1 (about 62%), linoleic C18:2 (20%), linolenic C18:3 (10%) and eicosenoic C20:1 (1%) [5–7], in addition to bioactive compounds and antioxidants, important components of functional food including natural biofortification [8, 9]. Such oil is valuable as salad oil and salad dressing. However, due to the presence of a large amount of polyunsaturated fatty acids, the rapeseed oil is less suitable for deep frying and industrial purposes, as there is a possibility of the formation of trans-fatty acids and oil oxidation during high-temperature treatment or long-term storage [10]. As functional oils with an enhanced nutritional value are of great importance [11], multiple efforts are undertaken to improve the oil quality for specific purposes of the highly competitive global plant oil market.

The physical and chemical properties of canola oil depend on the composition of seed oil fatty acids [5, 12, 13], especially the major C18 mono- and polyunsaturated fatty acids [10]. High content of oleic acid in rapeseed oil is desirable for its long shelf life and stability at high temperature [14], which makes it an optimal raw material for biodiesel production [15]. Polyunsaturated linolenic acid (C18:3) is prone to oxidation and unstable during frying and its reduced level in rapeseed oil is required for long storage [16] and also optimal for deep-fat frying [17], thus making it a target for fast-food producers.

In addition to its food-use, rapeseed oil is also applied as a raw material for the production of lubricants, pharmaceuticals, tensides, biodegradable plastics, and varnishes [18]. Moreover, in recent years, there has been an increasing interest in the use of plant oils with high oleic acid and low linolenic acid as a renewable raw material in the production of biofuels [18, 19]. Renewable energy sources are of significance [20, 21] and are gaining huge support for use in energy sectors [11] due to the need for lowering the emission of carbon dioxide which has increased mainly as a result of the use of fossil fuels as an energy source, particularly by the transportation sector. The increasing demand for biodiesel reinforces an increase in the production and acreage of rapeseed which benefits local farmers and also oil factories. In the European Union, over 11 MMT of biodiesel was produced in 2016; it is noteworthy that the seed meal remaining after biodiesel processing can be used as feedstock and a natural resource of brassinosteroids and plant hormones, due to its beneficial properties for both humans and plants [22]. However, further efforts in improving *Brassica* oilseeds to produce oil with high oleic and low linolenic acids, so–called high stable oil [1], with a special focus on reducing the content of linolenic acid to prevent oil oxidation [23] are needed.

Improved long storage and thermo-stability of canola oil has been one of the special goals of breeding programs for about two decades [11, 12, 24–26]. In 2007, the HOLL (high oleic low linolenic) canola oil was defined by Maher et al., in new spring canola varieties in Australia, as the oil characterized by more than 65% of oleic acid and less than 3% of linolenic acid [27]. Subsequently, Canadian and Australian open pollinated as well as hybrid spring HOLL varieties were characterized by 68% of oleic acid and 3% of linolenic acid in seed oil. They were released by Cargill and reported by Salisbury et al. [http://www.australianoilseeds.com/__data/assets/pdf_file/0005/7079/salisbury,_phil_00150_.pdf]. In Europe, the newly registered winter HOLL hybrid varieties were characterized by about 80% of oleic and less than 3% of linolenic acid in seed oil [https://www.dsv-seeds.com/oilseed-rape/winter-osr/holl-oilseed-rape.html; https://www.weloveholl.co.uk/]. A current standard industry-wide definition of so-called HOLL (or HOLLi) canola includes varieties producing oil with more than 75% of oleic acid and less than 3% of linolenic acid. It was defined by Barth [17], regarding nutritional value of vegetable oils in a case of their application for frying. Friedt and Snowdon [26] also stated that oil produced by so-called HOLL canola varieties is characterized by significantly higher oxidative stability while frying due to a strong reduction of trans-fatty acids formation, resulting from the low amount of linolenic acid (C18:3) in seed oil [28].

Initially, HOLL rapeseed genotypes, including breeding lines, were developed by chemical mutagenesis using ethyl methanesulfonate (EMS) [7, 13, 29–32]. Subsequent selection of HOLL mutant genotypes in breeding programs by phenotyping using gas–liquid chromatography has been laborious and time-consuming due to the fact that the composition of fatty acids in seed oil is highly influenced by solar radiation and temperature [33] which makes it difficult to reliably compare genotypes from different experiments. A genetic–based approach to genotype selection seems more promising for breeding programs. Examples of successful approaches exploit, e.g., fatty acid desaturases FAD2 and FAD3, which are involved in the formation of oleic and linolenic acid, respectively [5, 6, 34]. QTL genetic mapping using several DNA markers, such as RAPD, AFLP, SSR, and SNP, revealed the loci linked to the content of oleic and linolenic acid on the A04 and C10 chromosomes of *B*. *napus* [13, 35, 36]. Allele-specific DNA markers were identified for an effective selection of homozygous mutated genotypes that can produce seed oil with changed content of oleic and linolenic acid in breeding programs. Restriction site generating–polymerase chain reaction (RG-PCR) assay was developed by Barret et al. [37] for the selection of low linolenic (LL) *B*. *napus* genotypes derived from the ‘Oro’ LL mutant developed by Rakow et al. [38], CAPS markers were developed by Falentin et al. [39] for determining the high oleic (HO) genotype, and Illumina qPCR-based assay was developed by Hu et al. [30] for the detection of both mutated *FAD2* and *FAD3* alleles as well as SNaPshot by Mikolajczyk et al. [40] for the detection of *FAD3*, and SNP markers by Yang et al. [13] for the detection of allelic variation of *FAD2* and *FAD3* genes. Such functional markers are highly useful for the direct selection of desirable *fad2* and *fad3* mutations in marker-assisted trait introgression and breeding of HOLL-type canola.

In this paper we report the development of HOLL winter oilseed rape (WOSR) breeding lines of good agricultural value. After combining two breeding programs, 24 genotypes of different origin and properties were selected, grown in four environments and characterized through plant phenotyping and DNA marker analysis. In this work, we analyse the results obtained from the field experiments and identify genotypes with the most favorable set of traits, in particular seed oil fatty acid composition, good yield and low glucosinolates content. We also investigate trait stability by analyzing genotype–environment interaction. The selected desired genotypes make a valuable starting material to be used for further breeding.

## Materials and methods

### Plant material

In this study, we combined two independent breeding programs to obtain WOSR breeding lines characterized by high oleic and low linolenic acid content in seed oil in addition to low content of glucosinolates (LGLS) in seed meal, as well as good agricultural value including improved seed yield and wintering. One of the breeding programs aimed at the development of HOLL mutant recombinants by crossing HOmut (≥75%) and LLmut (≤3%) WOSR mutant lines obtained previously by Spasibionek [31] *via* chemical mutagenesis using ethyl methanesulfonate (EMS). The mutant HOmutLLmut F5 inbred recombinants obtained could not be used directly for pre-breeding as they were of low agricultural value characterized by low yield, low wintering, and increased GLS content in seed meal. Another breeding program aimed at recurrent selection of HO- and LGLS-type domestic WOSR accessions of good agricultural value [41]. Ten high oleic and low glucosinolates (HOLGLS) breeding lines were selected in several independent field trials. The selected lines were of HO type, which can produce oil characterized by about 78% of oleic acid (C18:1) in seed oil. In addition, their seed meal had extremely low GLS content (5–10 μmoles/g of seeds) and their seed yield was up to 48.7 dt ha^-1^ [41].

**Fig 1:**
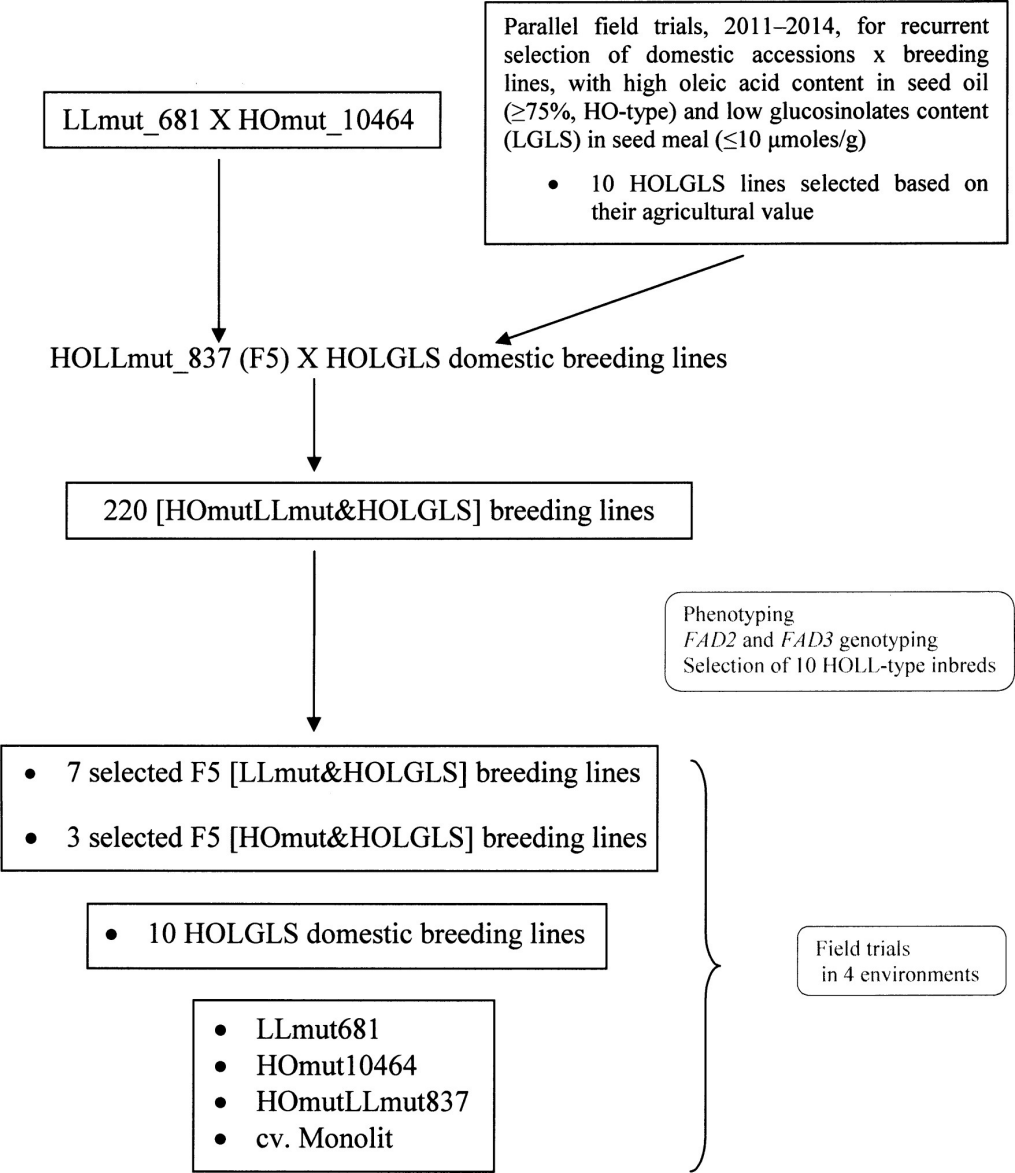
Breeding scheme used in this study. HOmut and LLmut, high oleic and low linolenic WOSR mutant lines obtained by chemical mutagenesis (EMS); HOLLmut, recombinant line obtained by crossing HOmut and LLmut lines; *FAD2* and *FAD3* genotyping with the use of specific DNA markers for monitoring mutated alleles of FAD2 and FAD3 fatty acid desaturase genes, responsible for biosynthesis of oleic and linolenic acids in seed oil, respectively.

To combine the two programs, an F5 inbred mutant recombinant (HOmutLLmut_837) was crossed with the 10 selected HOLGLS domestic breeding lines to develop new HOLL-type recombinants of good agricultural value. The breeding scheme is presented in Fig 1. From 220 analyzed recombinants, ten breeding lines were selected for detailed assessment in field trials together with their ancestral genotypes. Thus, in this study, plant material from all stages of the breeding program is investigated: ten HOLGLS domestic breeding lines, seven LLmut&HOLGLS and three HOmut&HOLGLS inbreds, one HOLLmut mutant recombinant line in addition to its parental mutant lines, HOmut and LLmut, and also cv. Monolit, a high-yielding Polish WOSR cultivar, as a reference (Table 1). The HOLLmut_837 line was an F5 inbred derived from crossing between a low linolenic (≤3%) mutant (LLmut_681) breeding line [31] and a high oleic (≥75%) mutant (HOmut_10464) line [31] (Fig 1; Table 1).

**Table 1:** Plant material used in this study.

Genotype	Parent	Gen.	Reference
LLmut_681	Mut681	M8	Spasibionek, 2006
HOmut_10464	Mut10464	M8	Spasibionek, 2006
HOmutLLmut_837	Mut10646xMut681	F5	This study
HOLGLS_480	HO-type and LGLS accession 5	F4	Spasibionek et al., 2016
HOLGLS_481	HO-type and LGLS accession 4	F4	Spasibionek et al., 2016
HOLGLS_490	HO-type and LGLS accession 8	F4	Spasibionek et al., 2016
HOLGLS_519	HO-type and LGLS accession 6	F5	Spasibionek et al., 2016
HOLGLS_520	HO-type and LGLS accession 7	F5	Spasibionek et al., 2016
HOLGLS_535	HO-type and LGLS accession 3	F5	Spasibionek et al., 2016
HOLGLS_543	HO-type and LGLS accession 9	F7	Spasibionek et al., 2016
HOLGLS_550	HO-type and LGLS accession 2	F9	Spasibionek et al., 2016
HOLGLS_561	HO-type and LGLS accession 10	F9	Spasibionek et al., 2016
HOLGLS_593	HO-type and LGLS accession 1	F9	Spasibionek et al., 2016
LLmut&HOLGLS_440	HOmutLLmut_837xHOLGLS_550	F5	This study
LLmut&HOLGLS_878	HOmutLLmut_837xHOLGLS_480	F5	This study
LLmut&HOLGLS_880	HOmutLLmut_837xHOLGLS_593	F5	This study
LLmut&HOLGLS_882	HOmutLLmut_837xHOLGLS_535	F5	This study
LLmut&HOLGLS_888	HOmutLLmut_837xHOLGLS_543	F5	This study
LLmut&HOLGLS_899	HOmutLLmut_837xHOLGLS_519	F5	This study
LLmut&HOLGLS_902	HOmutLLmut_837xHOLGLS_561	F5	This study
HOmut&HOLGLS_850	HOmutLLmut_837xHOLGLS_520	F5	This study
HOmut&HOLGLS_852	HOmutLLmut_837xHOLGLS_490	F5	This study
HOmut&HOLGLS_873	HOmutLLmut_837xHOLGLS_481	F5	This study
Monolit	Monolit, Polish cultivar, HO-type	cv.	http://www.coboru.pl

Gen., generation; LLmut, low-linolenic acid mutant genotype; HOmut, high-oleic acid mutant genotype; Mut, mutant genotype; LGLS, low-glucosinolate genotype; HOLGLS, high-oleic acid and low-glucosinolate genotype.

### Field trials and phenotyping

The lines were assessed in two locations: experimental station in Borowo (B), 52° 7' 12" N, 16° 47' 19" E, Greater Poland, on sandy soil and in Lagiewniki (L), 51° 45' 40" N, 17° 14' 13" E, Greater Poland, on sandy clay soil. The trials were conducted in two growing seasons: 2015/2016 (16) and 2016/2017 (17), arranged in four replications for each accession in a randomized complete block design and a plot size of 12 m^2^, including the core plot for harvest of 10 m^2^. The sowing dates in Borowo and Lagiewniki were, respectively: 25^th^ and 29^th^ August 2015, and 27^th^ and 28^th^ August 2016, with a sowing density of 70 seeds/m^2^. Seeds were harvested on 26^th^ and 27^th^ July 2016 and 22^nd^ July and 1^st^ August 2017, respectively, at seed maturity stage. Single-phase harvest was performed from core plots by plot combine harvester. Full chemical plant protection was applied in addition to other standard agronomical practices un the area.

**Fig 2:**
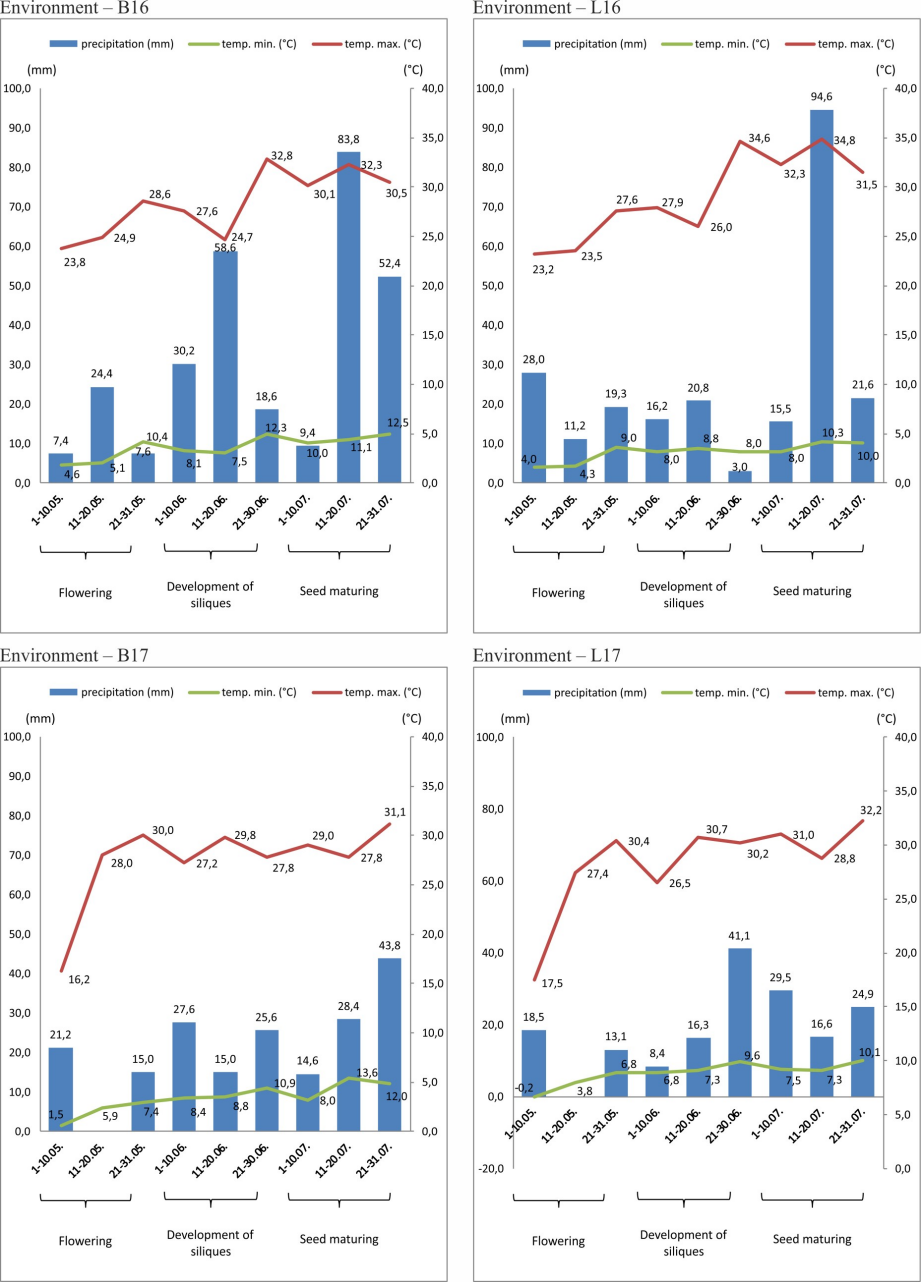
Weather conditions during flowering, development of siliques and seed maturing. B16 –Borowo, 2016; L16 –Lagiewniki, 2016; B17 –Borowo, 2017 L17 –Lagiewniki, 2017; blue color, rainfall [mm]; green and red, minimum and maximum temperature [°C], respectively.

Weather conditions varied moderately during growing seasons 2015/2016 and 2016/2017. Monthly means of temperature and precipitation, from August 2015 to July 2017, are included in S1 Table. Diagrams showing 10–day rainfall, in addition to minimum and maximum temperatures from the beginning of May (flowering) till the ending of July (harvest) in 2016 and 2017 in both locations (Borowo and Lagiewniki) are presented in Fig 2.

Phenotyping was performed for seed yield (dt ha^-1^), wintering (%), oil content of seed (% of dry seed weight), GLS content of seed (μmoles g^-1^ of seeds), and content of C18 unsaturated fatty acids in seed oil (C18:1 and C18:3; %). Wintering percent was measured by calculating the proportion of the number of plants in one row (3 m^2^) before and after winter.

Biochemical analysis of fatty acid composition in seed oil and GLS content in seed meal was done using a Hewlett Packard Agilent Technologies 6890N Network GC System. For GLS analysis, desulfoglucosinolates silyl derivatives were used [42, 43], and the European Commission CRM366 standard of 12.1 μmoles g^-1^ of seeds with a tolerance limit of 0.8 μmoles g^-1^ of seeds was applied. The seed oil content was measured using a broadband mass NMR spectroscopy analyzer (Newport Instruments Ltd.) [44].

### Statistical analysis

Statistical analysis of the phenotypic traits aimed at assessing the effects of genotypes and environments with respect to the observed traits, investigating the stability of oleic and linolenic acid content, and identifying genotypes with most favorable phenotypes. In the analyses, the combinations of year and location were regarded as independent environments. Significance of the effects of genotype, environment and their interaction was assessed by analysis of variance (ANOVA). Genotypic means, variances, and least significant differences at two significance levels (0.05 and 0.01) for all traits were obtained in Tukey multiple comparison test. Computations were performed in R statistical software. Assessment of accessions’ stability by means of analysis of the genotype-environment interaction (i.e., estimation of main and interaction effects for genotypes for C18:1 and C18:3 acid content as well as their visualization as biplots) was performed in SERGEN [45], according to the methodology developed by Kaczmarek [46] and Calinski et al. [47].

### Total DNA extraction and genotyping of allelic variants of *FAD2* and *FAD3* desaturase genes

Genomic DNA was isolated from young leaves using the modified CTAB method [48], as described by Mikolajczyk et al. [49]. The quality and quantity of DNA samples were estimated on 0.8% agarose gels using 50 ng of undigested lambda DNA as a reference, and by A_260_ and A_280_ UV absorbance measurements [49]. For monitoring the mutated and wild-type alleles of the functional *BnA*.*FAD2* desaturase gene, in the A genome of *B*. *napus* [50], involved in the synthesis of oleic acid, allele-specific CAPS markers [39] were used. PCR amplification products were analyzed using 1.4% agarose gel electrophoresis and scored as FAD2_A for wild-type and fad2_a for mutant alleles of *BnA*.*FAD2* desaturase gene. Wild-type and mutant alleles of FAD3 desaturase genes in the A and C genomes of *B*. *napus*, *BnA*.*FAD3* and *BnC*.*FAD3*, respectively, were scored as FAD3_A and FAD3_C for wild-type alleles and fad3_a and fad3_c for mutant alleles, using an allele-specific SNaPshot assay involving two steps: PCR amplification of short regions including possible mutation sites followed by microsequencing [40].

## Results

### Seed yield and wintering

In total, 24 accessions were assessed in multi-environment field trials and the reference cv. Monolit was characterized by the highest seed yield (34.89 dt ha^–1^). Twelve accessions revealed slightly lower but comparable seed yield of 28 dt ha^–1^ and more (regarding the calculated Tukey’s least significant difference (LSD) at the significance level of 0.01) (Table 2). Six of them were the new mutant recombinants: four LLmut&HOLGLS (440, 880, 882, and 899), and two HOmut&HOLGLS (850, and 873). The remaining six genotypes revealing favorable seed yield were HOLGLS accessions: 481, 519, 520, 535, 550, and 593. At the same time, the three mutant HOmut, LLmut, and HOmutLLmut genotypes were characterized by significantly low seed yield– 9.64, 9.99 and 12.19 dt ha^–1^, respectively. In addition, eight other genotypes: four HOLGLS (480, 490, 543, and 561), three LLmut&HOLGLS (878, 888, and 902), and one HOmut&HOLGLS (852) revealed seed yield higher than the mutant lines but lower than cv. Monolit (24.50–27.99 dt ha^–1^) (Table 2).

**Table 2:** Characteristics of the 24 lines tested in field trials conducted in four environments: Mean values of phenotypic traits and allelic variants of FAD2 and FAD3 desaturases in the A and C genomes of *B*. *napus*.

ID	Genotype	Seed yield (dt ha^-1^)	Wintering (%)	Oil cont.	C18:1	C18:3	GLS	FAD2_A/Chaplotype	FAD3_A/C haplotype
1	LLmut_681	9.99	37.85	43.82	68.31	2.34	10.68	wild/wild	mut/mut
2	HOmut_10464	9.64	66.21	44.44	78.63	6.49	13.84	mut/wild	wild/wild
3	HOLLmut_837	12.19	81.29	43.56	76.76	3.75	14.24	mut/wild	mut/mut
4	HOLGLS_480	27.34	82.01	45.89	78.59	6.73	8.66	wild/wild	wild/wild
5	HOLGLS_481	30.48	67.68	46.49	79.59	6.48	9.21	wild/wild	wild/wild
6	HOLGLS_490	27.53	64.96	47.38	76.52	7.18	7.44	wild/wild	wild/wild
7	HOLGLS_519	31.26	65.24	46.60	76.68	6.90	5.31	wild/wild	wild/wild
8	HOLGLS_520	30.50	59.35	48.39	76.94	7.01	9.08	wild/wild	wild/wild
9	HOLGLS_535	31.59	84.70	47.74	76.59	6.84	7.04	wild/wild	wild/wild
10	HOLGLS_543	26.59	74.40	44.01	77.54	7.54	5.34	wild/wild	wild/wild
11	HOLGLS_550	31.98	69.96	45.18	76.20	8.18	8.59	wild/wild	wild/wild
12	HOLGLS_561	24.50	63.05	44.75	75.32	8.10	9.89	wild/wild	wild/wild
13	HOLGLS_593	32.19	67.70	47.90	77.26	6.78	8.29	wild/wild	wild/wild
14	LLmut&HOLGLS_440	30.76	52.90	45.07	77.56	4.73	9.09	wild/wild	wild/mut
15	LLmut&HOLGLS_878	27.41	74.04	45.72	75.67	3.38	21.57	wild/wild	mut/mut
16	LLmut&HOLGLS_880	32.19	75.66	44.51	78.37	3.20	12.86	wild/wild	mut/mut
17	LLmut&HOLGLS_882	29.93	87.23	43.52	78.29	3.38	14.33	wild/wild	mut/mut
18	LLmut&HOLGLS_888	24.76	73.59	44.11	77.89	4.10	10.06	wild/wild	wild/mut
19	LLmut&HOLGLS_899	28.04	74.24	43.88	78.30	4.13	11.62	wild/wild	wild/mut
20	LLmut&HOLGLS_902	27.99	69.78	45.13	77.93	5.41	12.04	wild/wild	wild/mut
21	HOmut&HOLGLS_850	28.14	76.68	43.91	78.40	7.02	13.13	mut/wild	wild/wild
22	HOmut&HOLGLS_852	27.39	80.53	45.97	79.28	7.43	10.64	mut/wild	wild/wild
23	HOmut&HOLGLS_873	30.44	74.85	46.60	79.03	7.06	11.89	mut/wild	wild/wild
24	Monolit	34.89	81.30	45.56	64.46	8.15	12.16	wild/wild	wild/wild
Mean		26.99	71.05	45.42	76.67	5.93	10.71		
LSD 0.05		6.12	22.68	1.55	1.39	0.66	1.68		
LSD 0.01		6.89	25.73	1.75	1.56	0.74	1.89		

Oil cont. (%), seed oil content; C18:1 (%), oleic acid; C18:3 (%), linolenic acid; GLS, glucosinolates (μmoles g^-1^ of seeds); LSD, least significant difference.

The best wintering accession, LLmut&HOLGLS_882, was characterized by 87.23% of wintering. Five other genotypes, including the reference cv. Monolit (81.30%), revealed more than 80%: two HOLGLS (480 and 535), the HOmutLLmut_837, and the HOmut&LGLS_852. The LLmut_681 genotype revealed the lowest 37.85% of wintering. Two other accessions, LLmut&HOLGLS_440, and HOLGLS_520 showed low wintering percentage (less than 60%). The remaining 15 genotypes overwintered comparably to the best wintering accession, regarding the LSD 0.01 (Table 2).

### Seed oil content and quality

Seed oil content ranged between 43.52% (LLmut&HOLGLS_882) and 48.39% (the HOLGLS_520 domestic accession), whereas three HOLGLS domestic accessions (490, 535, and 593) revealed significantly high oil content of more than 46.64% (Table 2). Six other lines exceeded the reference cv. Monolit (45.56%): three HOLGLS accessions (480, 481, and 519), two HOmut&HOLGLS lines (852, and 873), and the LLmut&HOLGLS_878 line. Low seed oil content was observed for the HOmutLLmut_837 breeding line (Table 2).

There was a significant differentiation among the analyzed accessions with respect to the content of both oleic acid and linolenic acid in seed oil. The HOLGLS_481 domestic accession showed the highest C18:1 acid content (79.59%) and two HOmut&HOLGLS lines (852, and 873) revealed more than 79% as well. Six other lines revealed more than 78% of oleic acid in seed oil: the HOmut_10464 (78.63%), the HOLGLS_480 domestic accession (78.59%), the HOmut&HOLGLS_850 (78.40%), three LLmut&HOLGLS lines (880–78.37%, 882–78.29%, and 899–78.30%), as well as the LLmut&HOLGLS_880 (78.37%). Interestingly, the reference cv. Monolit showed the lowest oleic acid content of 64.46% (Table 2).

Regarding the content of linolenic acid in seed oil in the analyzed genotypes, the LLmut_681 line showed the lowest content of 2.34% and was used as a reference for the LL-type. Only three HOLL–type recombinant lines–LLmut&HOLGLS 878, 880, and 882 revealed less than 3.5% of linolenic acid. At the same time, they showed the homozygous low linolenic genotype (‘FAD3_A/C haplotype’ in Table 2).

The GLS content in seed meal was extremely low in the HOLGLS lines (including also the LLmut&HOLGLS_440 line), ranging from 5.34 to 9.21 μmoles g^-1^, as compared to cv. Monolit (12.16 μmoles g^-1^ of seeds). Moreover, all the genotypes were characterized by less than 15.00 μmoles g^-1^ of seeds (except for the LLmut&HOLGLS_878 line, which showed 21.57 μmoles g^-1^ of seeds) (Table 2).

### Genotype × environment interaction

Genotypes were evaluated in four environments (two years in two locations) by the variance analysis (Table 3) and the estimation of mean genotype and genotype by environment effects (Table 4). ANOVA for seed yield and seed quality traits (oil content, C18:1 and C18:3 fatty acids- content in seed oil and total seed GLS content in seed meal) revealed a statistically significant effects of genotype (G), environment (E), and the influence of the environment on genotypes (GxE) for all traits (Table 3).

**Table 3:** Variance estimates for seed yield, seed oil content and for seed quality.

Source of variation	Df	Mean Square Error				
		Yield	Seed oil content	C18:1	C18:3	GLS
**Environment (E)**	3	8942.71***	192.81***	151.86***	42.16***	203.05***
**Genotype (G)**	23	17030.43***	787.75***	4267.69***	1197.20***	4402.34***
**G × E**	69	6035.72***	306.08***	292.50***	56.18***	1251.86***
**Residuals**	288	6394.17	411.43	327.98	74.39	483.66

C18:1, oleic acid; C18:3, linolenic acid; GLS, glucosinolates

***significance level of at least 0.001.

**Table 4:** Genotype x environment interaction estimated for C18:1 and C18:3 fatty acids content in seed oil.

ID	Genotype	Oleic acid-C18:1			Linolenic acid-C18:3		
		Estimate of main effect	F-statistics for:		Estimate of main effect	F-statistics for:	
			main effect	interaction with environment		main effect	interaction with environment
1	LLmut_681	-8.36	102.22**	10.03**	-3.58	108.25**	8.94**
2	HOmut_10464	1.96	22.21*	2.54	0.56	3.41	7.05**
3	HOLLmut_837	0.09	0.02	5.30**	-2.18	119.82**	2.98*
4	HOLGLS_480	1.92	28.98**	1.87	0.80	22.70*	2.10
5	HOLGLS_481	2.92	176.22**	0.71	0.55	27.71**	0.83
6	HOLGLS_490	-0.15	0.09	3.91**	1.25	54.74**	2.14
7	HOLGLS_519	0.01	0.00	0.54	0.97	13.24**	5.36**
8	HOLGLS_520	0.27	0.46	2.38	1.08	229.42**	0.38
9	HOLGLS_535	-0.08	0.05	2.09	0.91	14.72**	4.22**
10	HOLGLS_543	-0.87	3.28	3.37*	1.61	82.63**	2.36
11	HOLGLS_550	-0.47	1.99	1.63	2.52	180.69**	2.11
12	HOLGLS_561	-1.35	25.10*	1.07	2.17	78.78**	4.50**
13	HOLGLS_593	0.59	2.18	2.32	0.85	8.63	6.33
14	LLmut&HOLGLS_440	0.89	19.69*	0.59	-1.20	59.98**	1.80
15	LLmut&HOLGLS_878	-1.00	3.67	4.02**	-2.55	1078.45**	0.45
16	LLmut&HOLGLS_880	1.70	20.79*	2.04	-2.43	352.54**	1.59
17	LLmut&HOLGLS_882	1.62	8.75	4.42**	-2.55	449.94**	1.09
18	LLmut&HOLGLS_888	1.22	15.46*	1.41	-1.83	314.96**	0.80
19	LLmut&HOLGLS_899	1.63	50.94*	0.77	-1.80	100.52**	2.44
20	LLmut&HOLGLS_902	1.26	1.32	17.53**	-0.52	1.12	18.33**
21	HOmut&HOLGLS_850	1.73	8.69	5.05*	1.90	16.45**	5.43**
22	HOmut&HOLGLS_852	2.61	25.09*	3.99**	1.50	50.31**	3.38*
23	HOmut&HOLGLS_873	2.36	73.88**	1.10	1.13	66.81**	1.43
24	Monolit	-12.21	202.88**	10.79**	2.22	180.39**	2.06
	Mean	75.82			6.17		
Critical value; α = 0.05			10.13	2.64		10.13	2.64
Critical value; α = 0.01			34.12	3.85		34.12	3.85

*significance level of at least 0.05

**significance level of at least 0.01.

Genotype × environment interaction for C18:1 and C18:3 fatty acids in seed oil was assessed by estimating the main G effects and G × E interaction and their significance. As shown in Table 4, out of 24 tested accessions ten genotypes, including the reference cv. Monolit, revealed a significant interaction with the environment regarding the content of oleic acid: low linolenic mutant LLmut_681 line, high oleic and low linolenic mutant recombinant HOLLmut_837, two of ten analyzed HOLGLS lines (490 and 543) and three of seven LLmut&HOLGLS recombinants (882, 878, and 902), in addition to two of the three analyzed HOmut&HOLGLS recombinants (850 and 852) (Table 4). With respect to C18:3 linolenic acid content, nine genotypes showed a significant interaction with the environment: low linolenic mutant LLmut_681 line, high oleic mutant HOmut_10464, high oleic and low linolenic mutant recombinant HOLLmut_837, three out of the 10 analyzed HOLGLS lines (519, 535, and 561) as well as–similarly to C18:1 oleic acid content–two of the three analyzed HOmut&HOLGLS recombinants: 850 and 852 (Table 4). At the same time, ten genotypes–five HOLGLS lines (480, 481, 520, 535, and 550), four LLmut&HOLGLS recombinants (440, 880, 888, and 899), and one HOmut&HOLGLS recombinant (873)–revealed no significant G × E interaction (Table 4), reflecting their stability.

**Fig 3:**
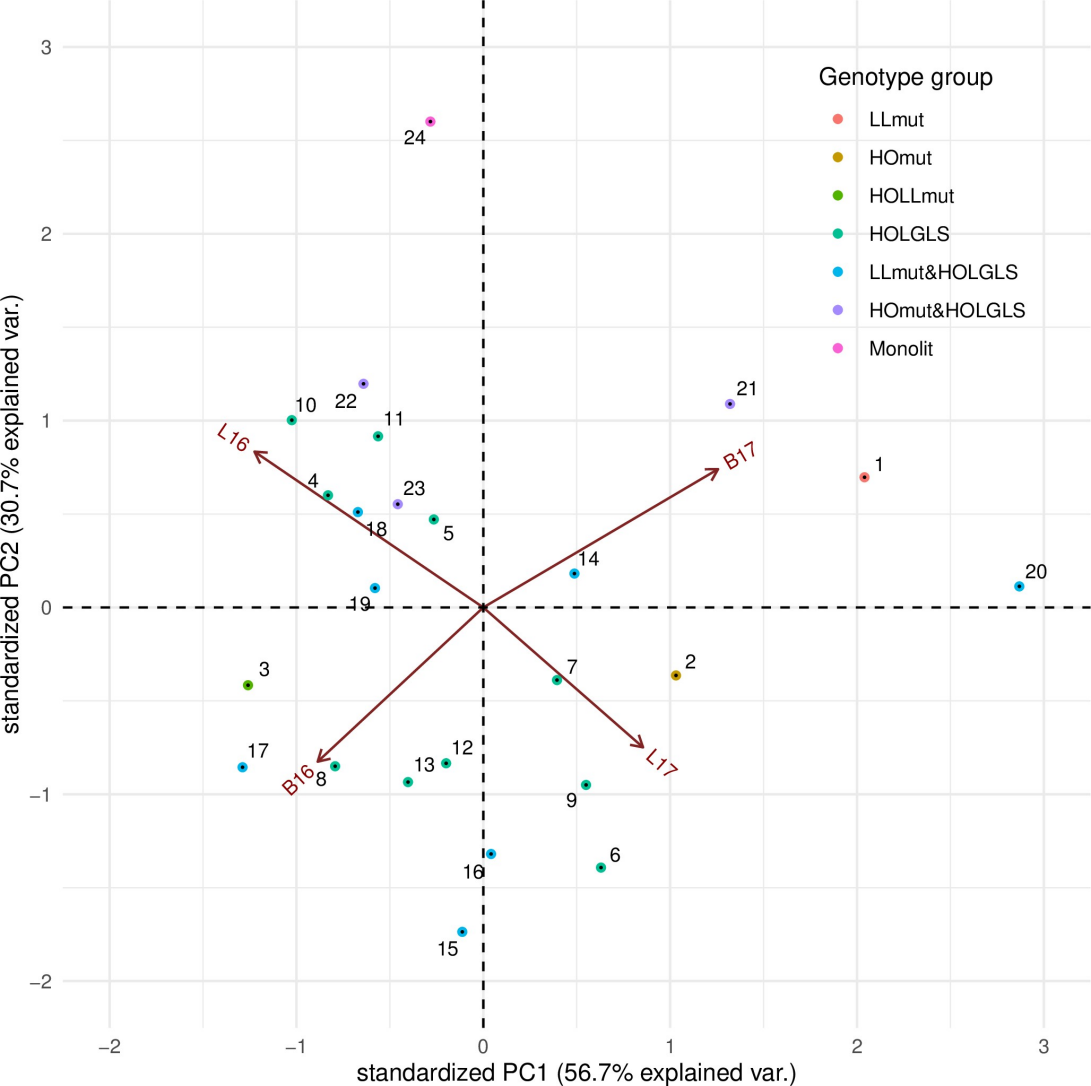
Biplot for G × E effect estimates of oleic acid content in genotypes in relation to the resultant environment. Genotypes are indicated by points and environments by lines. B—Borowo location; L—Lagiewniki location; 16–2015/2016 growing season; 17–2016/2017 growing season.

**Fig 4:**
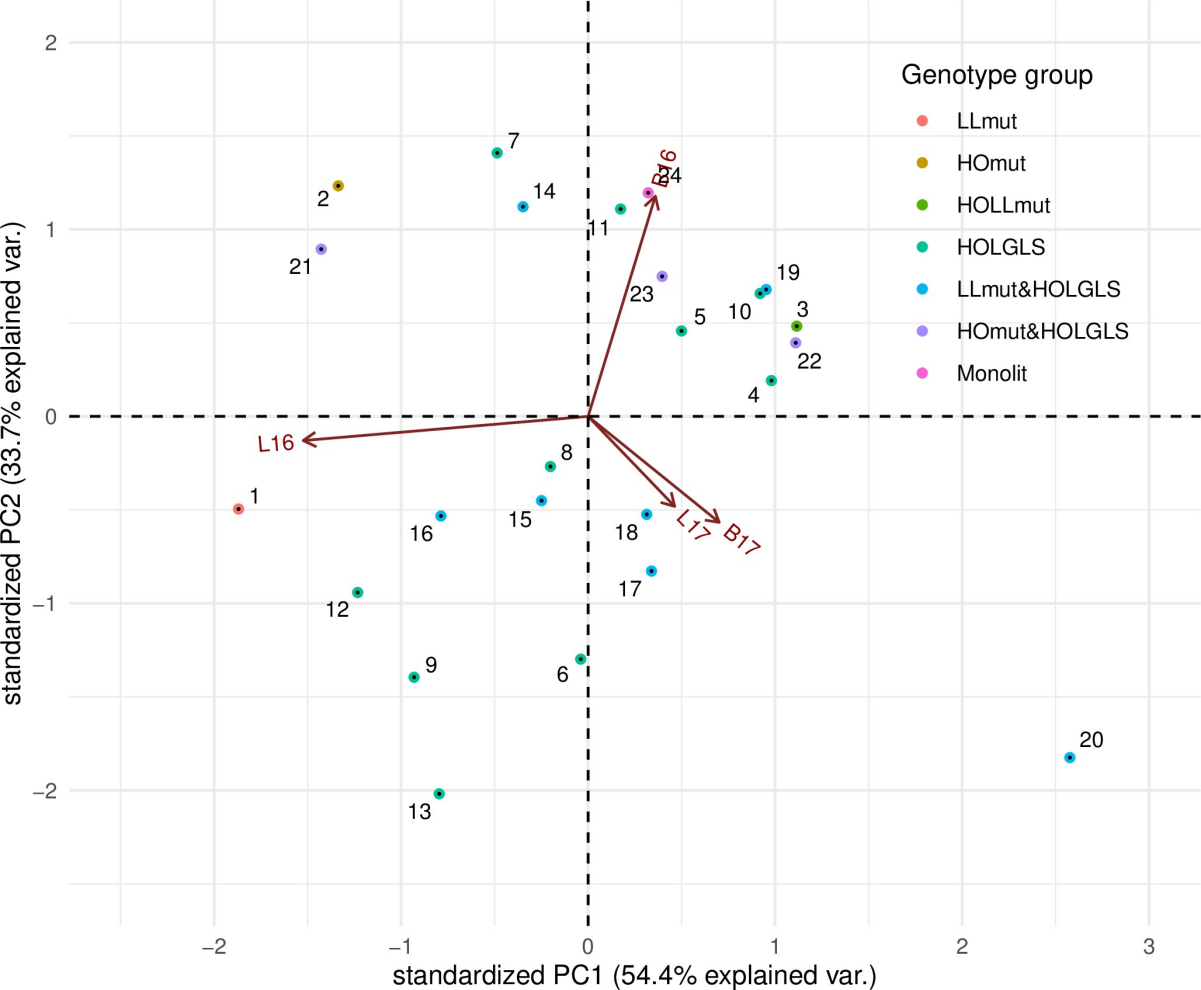
Biplot for G × E effect estimates of linolenic acid content in genotypes in relation to the resultant environment. Genotypes are indicated by points and environments by lines. B—Borowo location; L—Lagiewniki location; 16–2015/2016 growing season; 17–2016/2017 growing season.

To visualize the 24 analyzed genotypes with respect to the composition of C18:1 and C18:3 fatty acids in seed oil regarding the G × E interaction for the resultant environments (B16, B17, L16, and L17), a principal component analysis of the interaction effects was performed and shown graphically as biplots (Figs 3 and 4). The approach allowed us to identify the genotypes that show similar response patterns across environments and to group the environments that induce similar responses. The distances between the positions of genotypes and the origin reflect their interaction with the environment (cf. significant G × E effects in Table 4); the longer the distance, the more specific reaction of a genotype to the environmental conditions. The proximity of genotype positions to the environment vector indicates an above–average performance of the genotype in this environment. For the genotypes that demonstrate significant G × E interactions (Table 4), the biplot analysis reveals which environments contribute to the non–standard genotype performance. Small differences in response patterns against the average can also be observed for the genotypes identified as ‘stable’.

In the case of C18:1, we noted a group of genotypes with increased trait value in both B16 and L17. They were six HOLGLS domestic accessions: genotype 490 (ID6), 519 (ID7), 520 (ID8), 535 (ID9), 561 (ID12), 593 (ID13), and two LLmut&HOLGLS genotypes: 878 (ID15) and 880 (ID16). In B17 and L16, four HOLGLS accessions: 480 (ID4), 481 (ID5), 543 (ID10) and 550 (ID11) as well as the LLmut&HOLGLS_888 (ID18), HOmut&HOLGLS_852 (ID22) and HOmut&HOLGLS_873 (ID23) revealed similarly increased trait value. At the same time, environment B17 was specifically favourable for the low linolenic LLmut_681 (ID1) line, as well as for the LLmut&HOLGLS_902 (ID20) line, HOmut&HOLGLS_850 (ID21), and for the reference cv. Monolit (ID24). Environment L16 was favourable for the HOLGLS_543 (ID10), the HOmut&HOLGLS_852 (ID22), and for the reference cv. Monolit (ID24). Environment B16 was favourable for the HOmutLLmut_837 line (ID3), and also for the HOLGLS_490 domestic accession (ID6), and for two LLmut&HOLGLS lines: 878 (ID15) and 882 (ID17). Environment L17 was favourable for the LLmut_681 line (ID1), the HOLGLS_490 (ID6) domestic accession, and two LLmut&HOLGLS lines: 878 (ID15) and 902 (ID20). Interestingly enough, we noted opposing effects on C18:1 for locations in different years: L16 vs L17 and B16 vs B17.

For C18:3, we noted a group of genotypes with decreased trait value in B16. They were two HOLGLS domestic accessions: 490 (ID6) and 520 (ID8), and three LLmut&HOLGLS lines: 880 (ID16), 882 (ID17), and 888 (ID18). In addition, the LLmut_681 line (ID1) and two HOLGLS domestic accessions 535 (ID9) and 561 (ID12) revealed significantly decreased trait value in B16 (against L16, L17 and B17). A group of genotypes revealed decreased trait value in L16. They were: the LLmut_681 genotype (ID3), three HOLGLS domestic accessions: genotype 480 (ID4), 481 (ID5), 543 (ID10), the LLmut&HOLGLS_988 line (ID19), and two HOmut&HOLGLS genotypes 852 (ID22), and 873 (ID23), whereas for LLmut_681 genotype and for the HOmut&HOLGLS genotype_852 (ID22) the difference was significant. Interestingly, HOmut_10464, HOLGLS_519 (ID7), and HOmut&HOLGLS_850 (ID21) showed significantly increased trait value in B16 and L16 and decreased in B17 and L17. Moreover, B17 and L17 contribute to similar measurements of C18:3, while B16 and L16 supply different results.

For one genotype, the LLmut&HOLGLS_902 (ID20), both locations in 2017 contributed to significant increase of both C18:1 and C18:3.

## Discussion

Due to the application of canola oil as a raw material in various industries [1], multifaceted research and breeding has been performed at the PBAI-NRI Department of Genetics and Breeding of Oilseed Crops, Poznan, for further improvement of the oil quality to meet the demands of the plant oil crop market. One of the selection goals was to develop starting materials for the breeding of HOLL-type *B*. *napus* cultivars. Our first HO and LL mutant lines [31] were characterized by low agricultural value which was not only due to the toxic effects of the chemical mutagenesis (EMS treatment) but also a result of continuous inbreeding of canola over generations [18, 51]. Therefore, domestic WOSR accessions of different genetic background and good agricultural value were included in our breeding programs using recurrent selection [44] and the obtained breeding lines were further crossed with HOLL mutant recombinants. As a result, several new recombinant lines characterized by the modified fatty acid composition in seed oil exhibited improved seed yield in field trials. As a spectacular example, the HO (over 79% of oleic acid in seed oil) cv. Polka was first assessed for variety value for cultivation and use (VCU) [52] and then added to the Research Centre for Cultivar Testing (COBORU) National List (NLT) of Agricultural Plant Varieties in 2018 (http://www.coboru.pl). Unfortunately, the selection procedure of the new recombinants with changed fatty acid composition in seed oil based on phenotypic analysis (gas liquid chromatography) was not efficient due to its high dependence on changing environmental conditions [52, 53]. Therefore, for an effective selection of LL genotypes, application of allele-specific SNaPshot assay [40] was crucial.

The HOLL mutant recombinant line used in this work included a HO mutant line bearing one point mutation in the *BnA*.*FAD2* desaturase gene [39]. The comparison of the sequences with the other published sequences of this gene [13, 30] revealed that the described allele possesses a novel, previously uncharacterized point mutation G to A resulting in a STOP codon in the *BnA*.*FAD2* gene localized on the chromosome A05 in the main functional locus described by other authors [13, 30, 54]. Our LL mutant genotype is characterized by two statistically important single nucleotide polymorphisms (SNPs): (1) a G to A transition in the 5’splice donor site of the sixth intron in the *bnaC*.*fad3* gene disrupting the proper gene expression, and (2) a C to T substitution in the third position of the sixth codon of the seventh exon in the *BnaA*.*fad3* gene, leading to amino acid substitution, which may influence protein folding decreasing its enzymatic activity [40].

Hu et al. [30] characterized the spring canola mutant HOLL DMS100 line. The authors mapped one major and one minor locus for *BnaA*.*FAD2* genes on A05 and A01 *B*. *napus* chromosomes, respectively, as well as two major loci for *BnaC*.*FAD3* genes on A04 and C05 chromosomes. In the *bnaA*.*fad2* gene, a C to T single nucleotide substitution leading to the TAG stop codon in the *bnaA*.*fad2* gene was identified. The mutation caused the premature termination of the open reading frame and was associated with high oleic acid content. In the *bnaC*.*fad3* gene, a G to A transition in the 5’ donor splice site of the sixth intron in the *bnaC*.*fad3* gene was associated with LL genotype [30]. The point mutation was the same as in the LL mutant genotype in our previous study [40]. In the HOLL mutant variety released by SW Hickory, Yang et al. [13] identified one deleted mutation associated with high oleic acid content in the *BnaA*.*FAD2*.*a* locus which was not reported previously. At the same time, the authors identified two mutated *FAD3* alleles for low linolenic acid content in SW Hickory in the *BnaA*.*FAD3*.*b* locus. The first one was a C to T substitution in exon 2 leading to a synonymous mutation, and the second was a C to T transition in exon 7, resulting in an amino acid substitution from arginine to cysteine. The latter was the same as identified previously in our mutant low-linolenic genotype [40]. Moreover, Hu et al. [30], Mikolajczyk et al. [40], and Yang et al. [13], independently identified one C to T point mutation in the 5’ splice site of the sixth intron in the *BnaC*.*FAD3*.*b* locus.

Analysis of G **×** E interaction revealed different behavior of the analyzed genotypes in the four environments. In both growing seasons, during germination (August) and rosette formation (September–November) mean monthly temperatures were higher than the adequate multiyear means, whereas mean monthly rainfalls were significantly lower (except for Borowo in August 2015). As a consequence, notable differences in rosette development before winter were observed in each environment. During winter, temperature and precipitation were favorable for plant overwintering in each environment. During spring vegetation (March–April) both temperature and rainfall varied among environments (S1 Table), however, it did not influence the proper stem elongation nor inflorescence emergence. During flowering (May) in 2017 there were short periods of lack of rainfall (May 11^th^– 20^th^) in both locations (B17 and L17), but this did not significantly influence the length of flowering period, nor negatively influenced the analyzed phenotypic traits, as compared to B16 and L16 (S2 and S3 Tables). During development of siliques (June), there was enormously high rainfall in Borowo in 2016 (B16) leading to early stem lodging as well as the to the delayed development of siliques. It might have caused the lower mean seed yield in B16, as compared to the B17 (S4 Table). At the same time, it did not significantly influence the mean oil content as well as C18:1 and C18:3 content in seed oil (S4 Table). During seed maturing and seed fill period (July), there was high rainfall in both locations in 2016, unlike in 2017. However, it did not substantially influence mean values of oil content, nor C18:1 and C13:3 fatty acid content in seed oil between B16 and B17, and L16 and L17. At the same time, there were significant differences in C18:1 and C18:3 fatty acid content for certain genotypes regarding one location and different years (S3 Table).

Different G **×** E interaction patterns revealed by particular breeding lines may result from their origin and different genetic background of domestic accessions used for crossing in breeding trials. In this work, we were looking for stable HOLL–type canola genotypes revealing good agricultural value. This is why the LLmut&HOLGLS_880 line is of our special interest and will be included in further breeding programs involving development of high yielding F1 hybrid varieties with improved biotic stress resistance. Homozygotes selection with the use of functional markers specific for mutated alleles of the FAD3 desaturase genes was necessary to maintain the low linolenic acid content in seed oil. It is in accordance with our previous results on determination of fatty acid composition in canola seed oil by mutated alleles of the *FAD3* genes in the A and C genomes of *B*. *napus* [55].

The development of HOLL-type breeding lines of *B*. *napus* remains one of the special selection goals worldwide [1], and our new HOLL recombinant genotypes make a valuable resource on the world oil crop market. It is noteworthy that two of the LLmut&HOLGLS recombinants, 880 and 882, were characterized by seed yield comparable to the reference cv. Monolit, in addition to a high percentage of wintering as well as high content of oleic acid and low content of linolenic acid, thus revealing their HOLL phenotype (Table 2). At the same time, 880 was stable in different environments for both fatty acid content traits, while 882 for C18:3 (Table 4). Moreover, according to the genotyping results these two recombinant lines revealed wild-type homozygous alleles of FAD2 desaturase gene involved in the synthesis of oleic acid and at the same time, mutant homozygous alleles of FAD3 desaturase genes involved in the synthesis of linolenic acid (Table 2). The newly developed *B*. *napus* lines are genetic resources of seed oil with changed fatty acid composition and thus they can support the demands of the world oil crops market regarding the use of plant oils for deep frying and preparing healthy food as well as in biodiesel production.

## Supporting information

S1 Table Mean temperatures and precipitation in four environments. (DOCX)

S2 Table Flowering period of the analyzed genotypes in four environments. (DOCX)

S3 Table Percentage of oleic (C18:1) and linolenic (C18:3) acid content in seed oil of the analyzed genotypes in four environments. (DOCX)

S4 Table Characteristics of field trials in four environments. (DOCX)
