# Supplementary material for: Marker assisted selection of new high oleic and low linolenic winter oilseed rape (Brassica napus L.) inbred lines revealing good agricultural value
Source: PLoS One. 2020 Jun 4;15(6):e0233959. doi: 10.1371/journal.pone.0233959 (PMC7272079; doi:10.1371/journal.pone.0233959)
Supplement: S1 Table — (DOCX) [file pone.0233959.s001.docx]

**S1 Table. Mean temperatures and precipitation in four environments.**

| **Growth stages** | **Month** | | **Temperature [^o^C]** | | | | **Precipitation [mm]** | | | |
| --- | --- | --- | --- | --- | --- | --- | --- | --- | --- | --- |
|  |  |  | **B16** | **L16** | **B17** | **L17** | **B16** | **L16** | **B17** | **L17** |
| Germination | August | Monthly mean/sum | 22,0 | 22,8 | 18,0 | 18,1 | 60,6 | 30,6 | 31,6 | 22,7 |
|  |  | Multiyear mean 1957-2017 | 17,7 | 18,0 | 17,7 | 18,0 | 63,7 | 68,6 | 63,7 | 66,6 |
| Rosette development | September | Monthly mean/sum | 15,0 | 15,1 | 16,0 | 16,8 | 29,8 | 31,3 | 8,2 | 10,4 |
|  |  | Multiyear mean 1957-2017 | 13,4 | 13,6 | 13,4 | 13,7 | 47,7 | 42,2 | 47,7 | 43,0 |
|  | October | Monthly mean/sum | 8,0 | 7,9 | 8,0 | 8,2 | 16,6 | 19,0 | 95,2 | 105,6 |
|  |  | Multiyear mean 1957-2017 | 8,7 | 8,8 | 8,7 | 8,8 | 32,3 | 38,0 | 32,3 | 38,4 |
|  | November | Monthly mean/sum | 6,0 | 6,0 | 3,0 | 3,3 | 44,4 | 46,6 | 30,0 | 33,7 |
|  |  | Multiyear mean 1957-2017 | 3,7 | 4,0 | 3,7 | 3,9 | 42,8 | 40,1 | 42,8 | 39,7 |
| Winter dormancy | December | Monthly mean/sum | 6,0 | 5,3 | 2,0 | 1,4 | 22,1 | 26,3 | 24,4 | 38,9 |
|  |  | Multiyear mean 1957-2017 | 0,4 | 0,1 | 0,4 | 0,1 | 42,1 | 39,3 | 42,1 | 38,5 |
|  | January | Monthly mean/sum | -2,0 | -1,9 | -1,0 | -3,0 | 44,4 | 39,8 | 44,2 | 19,4 |
|  |  | Multiyear mean 1957-2017 | -1,1 | -1,5 | -1,1 | -1,5 | 28,6 | 32,3 | 28,6 | 32,6 |
|  | February | Monthly mean/sum | 4,0 | 3,5 | 3,2 | 0,6 | 29,2 | 51,1 | 15,3 | 37,8 |
|  |  | Multiyear mean 1957-2017 | -0,4 | -0,3 | -0,4 | -0,3 | 28,7 | 32,0 | 28,7 | 29,0 |
| Stem elongation, inflorescence emergence | March | Monthly mean/sum | 4,0 | 4,0 | 7,0 | 6,4 | 55,0 | 43,5 | 23,6 | 22,6 |
|  |  | Multiyear mean 1957-2017 | 3,4 | 3,2 | 3,4 | 3,3 | 29,5 | 33,0 | 29,5 | 33,4 |
|  | April | Monthly mean/sum | 9,0 | 8,4 | 8,0 | 7,6 | 39,8 | 49,4 | 27,6 | 43,9 |
|  |  | Multiyear mean 1957-2017 | 7,5 | 8,3 | 7,5 | 8,3 | 31,2 | 31,4 | 31,2 | 31,7 |
| Flowering | May (1-10) | min | 4,6 | 4,0 | 1,5 | -0,2 | 7,4 | 28,0 | 21,2 | 18,5 |
|  |  | max | 23,8 | 23,2 | 16,2 | 17,5 |  |  |  |  |
|  | May (11-20) | min | 5,1 | 4,3 | 5,9 | 3,8 | 24,4 | 11,2 | 0,0 | 0,0 |
|  |  | max | 24,9 | 23,5 | 28,0 | 27,4 |  |  |  |  |
|  | May (21-31) | min | 10,4 | 9,0 | 7,4 | 6,8 | 7,6 | 19,3 | 15,0 | 13,1 |
|  |  | max | 28,6 | 27,6 | 30,0 | 30,4 |  |  |  |  |
|  |  | Monthly mean/sum | 15,0 | 15,2 | 12,0 | 14,1 | 39,4 | 58,5 | 36,2 | 31,6 |
|  |  | Multiyear mean 1957-2017 | 13,1 | 13,6 | 13,3 | 13,6 | 47,4 | 56,5 | 47,4 | 56,1 |
| Development of siliques | June (1-10) | min | 8,1 | 8,0 | 8,4 | 6,8 | 30,2 | 16,2 | 27,6 | 8,4 |
|  |  | max | 27,6 | 27,9 | 27,2 | 26,5 |  |  |  |  |
|  | June (11-20) | min | 7,5 | 8,8 | 8,8 | 7,3 | 56,8 | 20,8 | 15,0 | 16,3 |
|  |  | max | 24,7 | 26,0 | 29,8 | 30,7 |  |  |  |  |
|  | June (21-30) | min | 12,3 | 8,0 | 10,9 | 9,6 | 18,6 | 3,0 | 25,6 | 41,1 |
|  |  | max | 32,8 | 34,6 | 27,8 | 30,2 |  |  |  |  |
|  |  | Monthly mean/sum | 18,0 | 18,8 | 17,2 | 18,4 | 105,4 | 40,0 | 68,2 | 65,8 |
|  |  | Multiyear mean 1957-2017 | 16,2 | 16,8 | 16,3 | 16,9 | 62,8 | 65,4 | 62,8 | 65,4 |
| Seed maturing | July (1-10) | min | 10,0 | 8,0 | 8,0 | 7,5 | 9,4 | 15,5 | 14,6 | 29,5 |
|  |  | max | 30,1 | 32,3 | 29,0 | 31,0 |  |  |  |  |
|  | July (11-20) | min | 11,1 | 10,3 | 13,6 | 7,3 | 83,8 | 94,6 | 28,4 | 16,6 |
|  |  | max | 32,3 | 34,8 | 27,8 | 28,8 |  |  |  |  |
|  | July (21-31) | min | 12,5 | 10,0 | 12,0 | 10,1 | 52,4 | 21,6 | 43,8 | 24,9 |
|  |  | max | 30,5 | 31,5 | 31,1 | 32,2 |  |  |  |  |
|  |  | Monthly mean/sum | 19,0 | 19,6 | 20,0 | 18,9 | 145,6 | 131,7 | 86,8 | 71,0 |
|  |  | Multiyear mean 1957-2017 | 18,5 | 18,6 | 18,5 | 18,6 | 76,9 | 81,9 | 76,9 | 81,9 |

B16, Borowo 2015/2016; L16, Lagiewniki 2015/2016; B17, Borowo 2016/2017; L17, Lagiewniki 2016/2017
