# Supplementary material for: Marker assisted selection of new high oleic and low linolenic winter oilseed rape (Brassica napus L.) inbred lines revealing good agricultural value
Source: PLoS One. 2020 Jun 4;15(6):e0233959. doi: 10.1371/journal.pone.0233959 (PMC7272079; doi:10.1371/journal.pone.0233959)
Supplement: S2 Table — (DOCX) [file pone.0233959.s002.docx]

**S2 Table. Flowering period of the analyzed genotypes in four environments.**

| **ID** | **Genotype** | **B16** | | | **L16** | | | **B17** | | | **L17** | | | **1 - Flowering start** | | | **2 - Flowering end** | | | **3 - Flowering days** | | |
| --- | --- | --- | --- | --- | --- | --- | --- | --- | --- | --- | --- | --- | --- | --- | --- | --- | --- | --- | --- | --- | --- | --- |
|  |  | **1** | **2** | **3** | **1** | **2** | **3** | **1** | **2** | **3** | **1** | **2** | **3** | **Mean** | **Min.** | **Max.** | **Mean** | **Min.** | **Max.** | **Mean** | **Min.** | **Max.** |
| 1 | LLmut_681 | 120 | 154 | 34 | 114 | 154 | 40 | 111 | 151 | 40 | 108 | 153 | 45 | 113 | 108 | 120 | 153 | 151 | 154 | 40 | 34 | 45 |
| 2 | HOmut_10464 | 121 | 149 | 28 | 126 | 155 | 29 | 128 | 154 | 26 | 130 | 154 | 24 | 126 | 121 | 130 | 153 | 149 | 155 | 27 | 24 | 29 |
| 3 | HOmutLLmut_837 | 121 | 149 | 28 | 126 | 149 | 23 | 125 | 148 | 23 | 126 | 152 | 26 | 124 | 121 | 126 | 149 | 148 | 152 | 25 | 23 | 28 |
| 4 | HOLGLS_480 | 112 | 144 | 32 | 121 | 156 | 35 | 112 | 146 | 34 | 116 | 149 | 33 | 115 | 112 | 121 | 149 | 144 | 156 | 33 | 32 | 35 |
| 5 | HOLGLS_481 | 119 | 150 | 31 | 123 | 156 | 33 | 113 | 146 | 33 | 117 | 150 | 33 | 118 | 113 | 123 | 150 | 146 | 156 | 32 | 31 | 33 |
| 6 | HOLGLS_490 | 121 | 152 | 31 | 125 | 153 | 28 | 120 | 147 | 28 | 121 | 152 | 31 | 122 | 120 | 125 | 151 | 147 | 153 | 29 | 28 | 31 |
| 7 | HOLGLS_519 | 112 | 143 | 31 | 124 | 152 | 28 | 112 | 146 | 35 | 119 | 150 | 31 | 116 | 112 | 124 | 148 | 143 | 152 | 31 | 28 | 35 |
| 8 | HOLGLS_520 | 117 | 148 | 31 | 125 | 150 | 25 | 112 | 147 | 35 | 118 | 151 | 33 | 118 | 112 | 125 | 149 | 147 | 151 | 31 | 25 | 35 |
| 9 | HOLGLS_535 | 122 | 153 | 31 | 125 | 153 | 28 | 117 | 144 | 28 | 121 | 147 | 26 | 121 | 117 | 125 | 149 | 144 | 153 | 28 | 26 | 31 |
| 10 | HOLGLS_543 | 111 | 136 | 25 | 119 | 150 | 31 | 111 | 144 | 34 | 114 | 147 | 33 | 113 | 111 | 119 | 144 | 136 | 150 | 31 | 25 | 34 |
| 11 | HOLGLS_550 | 121 | 146 | 25 | 125 | 153 | 28 | 116 | 144 | 29 | 117 | 147 | 30 | 119 | 116 | 125 | 147 | 144 | 153 | 28 | 25 | 30 |
| 12 | HOLGLS_561 | 121 | 150 | 29 | 125 | 156 | 31 | 123 | 147 | 24 | 125 | 151 | 26 | 123 | 121 | 125 | 151 | 147 | 156 | 28 | 24 | 31 |
| 13 | HOLGLS_593 | 118 | 142 | 24 | 124 | 152 | 28 | 111 | 147 | 35 | 118 | 151 | 33 | 118 | 111 | 124 | 148 | 142 | 152 | 30 | 24 | 35 |
| 14 | LLmut&HOLGLS_440 | 110 | 138 | 28 | 118 | 158 | 40 | 110 | 145 | 35 | 112 | 148 | 35 | 112 | 110 | 118 | 147 | 138 | 158 | 35 | 28 | 40 |
| 15 | LLmut&HOLGLS_878 | 120 | 152 | 32 | 120 | 150 | 30 | 113 | 146 | 33 | 117 | 151 | 34 | 117 | 113 | 120 | 150 | 146 | 152 | 32 | 30 | 34 |
| 16 | LLmut&HOLGLS_880 | 119 | 151 | 32 | 120 | 150 | 30 | 114 | 145 | 31 | 118 | 149 | 30 | 118 | 114 | 120 | 149 | 145 | 151 | 31 | 30 | 32 |
| 17 | LLmut&HOLGLS_882 | 118 | 150 | 32 | 121 | 152 | 31 | 114 | 146 | 32 | 118 | 149 | 31 | 118 | 114 | 121 | 149 | 146 | 152 | 31 | 31 | 32 |
| 18 | LLmut&HOLGLS_888 | 117 | 146 | 29 | 120 | 152 | 32 | 114 | 146 | 32 | 117 | 148 | 32 | 117 | 114 | 120 | 148 | 146 | 152 | 31 | 29 | 32 |
| 19 | LLmut&HOLGLS_899 | 111 | 143 | 32 | 119 | 150 | 31 | 111 | 145 | 34 | 115 | 148 | 34 | 114 | 111 | 119 | 147 | 143 | 150 | 33 | 31 | 34 |
| 20 | LLmut&HOLGLS_902 | 118 | 151 | 33 | 125 | 155 | 30 | 112 | 148 | 37 | 121 | 153 | 32 | 119 | 112 | 125 | 152 | 148 | 155 | 33 | 30 | 37 |
| 21 | HOmut&HOLGLS_850 | 118 | 146 | 28 | 121 | 153 | 32 | 112 | 147 | 35 | 117 | 148 | 31 | 117 | 112 | 121 | 148 | 146 | 153 | 31 | 28 | 35 |
| 22 | HOmut&HOLGLS_852 | 112 | 140 | 28 | 124 | 154 | 30 | 113 | 145 | 33 | 119 | 149 | 30 | 117 | 112 | 124 | 147 | 140 | 154 | 30 | 28 | 33 |
| 23 | HOmut&HOLGLS_873 | 121 | 153 | 32 | 125 | 156 | 31 | 115 | 145 | 31 | 121 | 147 | 27 | 120 | 115 | 125 | 150 | 145 | 156 | 30 | 27 | 32 |
| 24 | Monolit | 120 | 150 | 30 | 125 | 154 | 29 | 118 | 144 | 26 | 119 | 148 | 30 | 120 | 118 | 125 | 149 | 144 | 154 | 29 | 26 | 30 |

B16, Borowo 2016; L16, Lagiewniki 2016; B17, Borowo 2017; L17, Lagiewniki 2017.
