# Supplementary material for: Marker assisted selection of new high oleic and low linolenic winter oilseed rape (Brassica napus L.) inbred lines revealing good agricultural value
Source: PLoS One. 2020 Jun 4;15(6):e0233959. doi: 10.1371/journal.pone.0233959 (PMC7272079; doi:10.1371/journal.pone.0233959)
Supplement: S3 Table — (DOCX) [file pone.0233959.s003.docx]

**S3 Table. Percentage of oleic (C18:1) and linolenic (C18:3) acid content in seed oil of the analyzed genotypes in four environments.**

| **ID** | **Genotype** | **C18:1** | | | | | | | **C18:3** | | | | | | |
| --- | --- | --- | --- | --- | --- | --- | --- | --- | --- | --- | --- | --- | --- | --- | --- |
|  |  | **B16** | **B17** | **L16** | **L17** | **mean** | **min** | **max** | **B16** | **B17** | **L16** | **L17** | **mean** | **min** | **max** |
| 1 | LLmut_681 | 65,58 | 69,60 | 67,78 | 70,30 | 68,31 | 65,58 | 70,30 | 2,25 | 1,93 | 2,70 | 2,50 | 2,34 | 1,93 | 2,70 |
| 2 | HOmut_10464 | 77,58 | 79,13 | 77,90 | 79,93 | 78,63 | 77,58 | 79,93 | 7,13 | 6,55 | 6,60 | 5,70 | 6,49 | 5,70 | 7,13 |
| 3 | HOLLmut_837 | 76,13 | 74,80 | 78,30 | 77,80 | 76,76 | 74,80 | 78,30 | 4,38 | 4,28 | 2,68 | 3,68 | 3,75 | 2,68 | 4,38 |
| 4 | HOLGLS_480 | 77,85 | 77,93 | 79,90 | 78,70 | 78,59 | 77,85 | 79,90 | 7,23 | 7,25 | 5,73 | 6,70 | 6,73 | 5,73 | 7,25 |
| 5 | HOLGLS_481 | 78,35 | 79,10 | 80,58 | 80,33 | 79,59 | 78,35 | 80,58 | 7,03 | 6,83 | 5,70 | 6,38 | 6,48 | 5,70 | 7,03 |
| 6 | HOLGLS_490 | 76,48 | 76,45 | 75,38 | 77,78 | 76,52 | 75,38 | 77,78 | 7,00 | 7,70 | 6,73 | 7,28 | 7,18 | 6,73 | 7,70 |
| 7 | HOLGLS_519 | 75,70 | 76,45 | 76,60 | 77,95 | 76,68 | 75,70 | 77,95 | 7,68 | 6,38 | 6,50 | 7,05 | 6,90 | 6,38 | 7,68 |
| 8 | HOLGLS_520 | 77,18 | 75,85 | 77,30 | 77,45 | 76,94 | 75,85 | 77,45 | 7,20 | 7,38 | 6,60 | 6,85 | 7,01 | 6,60 | 7,38 |
| 9 | HOLGLS_535 | 76,23 | 76,55 | 75,83 | 77,75 | 76,59 | 75,83 | 77,75 | 6,53 | 7,20 | 6,83 | 6,80 | 6,84 | 6,53 | 7,20 |
| 10 | HOLGLS_543 | 76,73 | 76,93 | 79,18 | 77,33 | 77,54 | 76,73 | 79,18 | 8,20 | 7,78 | 6,53 | 7,65 | 7,54 | 6,53 | 8,20 |
| 11 | HOLGLS_550 | 75,18 | 75,90 | 77,45 | 76,28 | 76,20 | 75,18 | 77,45 | 8,93 | 8,08 | 7,50 | 8,23 | 8,18 | 7,50 | 8,93 |
| 12 | HOLGLS_561 | 75,20 | 74,68 | 75,25 | 76,15 | 75,32 | 74,68 | 76,15 | 7,93 | 8,40 | 8,23 | 7,85 | 8,10 | 7,85 | 8,40 |
| 13 | HOLGLS_593 | 77,58 | 76,63 | 77,10 | 77,73 | 77,26 | 76,63 | 77,73 | 6,25 | 7,25 | 6,73 | 6,90 | 6,78 | 6,25 | 7,25 |
| 14 | LLmut&HOLGLS_440 | 76,23 | 77,65 | 77,73 | 78,65 | 77,56 | 76,23 | 78,65 | 5,43 | 4,70 | 4,33 | 4,48 | 4,73 | 4,33 | 5,43 |
| 15 | LLmut&HOLGLS_878 | 75,38 | 74,23 | 75,48 | 77,60 | 75,67 | 74,23 | 77,60 | 3,50 | 3,73 | 3,00 | 3,30 | 3,38 | 3,00 | 3,73 |
| 16 | LLmut&HOLGLS_880 | 78,25 | 77,60 | 77,95 | 79,68 | 78,37 | 77,60 | 79,68 | 3,23 | 3,38 | 3,08 | 3,13 | 3,20 | 3,08 | 3,38 |
| 17 | LLmut&HOLGLS_882 | 78,43 | 76,53 | 79,28 | 78,95 | 78,29 | 76,53 | 79,28 | 3,43 | 3,88 | 2,73 | 3,50 | 3,38 | 2,73 | 3,88 |
| 18 | LLmut&HOLGLS_888 | 77,33 | 77,48 | 78,88 | 77,88 | 77,89 | 77,33 | 78,88 | 4,25 | 4,38 | 3,43 | 4,35 | 4,10 | 3,43 | 4,38 |
| 19 | LLmut&HOLGLS_899 | 77,80 | 77,70 | 79,08 | 78,63 | 78,30 | 77,70 | 79,08 | 4,80 | 4,40 | 3,10 | 4,20 | 4,13 | 3,10 | 4,80 |
| 20 | LLmut&HOLGLS_902 | 75,28 | 79,78 | 76,30 | 80,35 | 77,93 | 75,28 | 80,35 | 5,33 | 6,30 | 3,68 | 6,33 | 5,41 | 3,68 | 6,33 |
| 21 | HOmut&HOLGLS_850 | 76,23 | 79,60 | 78,35 | 79,43 | 78,40 | 76,23 | 79,60 | 7,50 | 6,63 | 7,13 | 6,83 | 7,02 | 6,63 | 7,50 |
| 22 | HOmut&HOLGLS_852 | 77,55 | 78,58 | 81,15 | 79,85 | 79,28 | 77,55 | 81,15 | 8,03 | 8,08 | 6,38 | 7,25 | 7,43 | 6,38 | 8,08 |
| 23 | HOmut&HOLGLS_873 | 77,85 | 78,43 | 80,20 | 79,63 | 79,03 | 77,85 | 80,20 | 7,70 | 7,43 | 6,33 | 6,78 | 7,06 | 6,33 | 7,70 |
| 24 | Monolit | 63,28 | 65,80 | 65,78 | 62,98 | 64,46 | 62,98 | 65,80 | 8,95 | 8,33 | 7,43 | 7,90 | 8,15 | 7,43 | 8,95 |
|  |  | **75,80** | **76,39** | **77,03** | **77,46** | **76,67** | **75,80** | **77,46** | **6,24** | **6,17** | **5,40** | **5,90** | **5,93** | **5,40** | **6,24** |

B16, Borowo 2015/2016; L16, Lagiewniki 2015/2016; B17, Borowo 2016/2017; L17, Lagiewniki 2016/2017
