# Supplementary material for: Marker assisted selection of new high oleic and low linolenic winter oilseed rape (Brassica napus L.) inbred lines revealing good agricultural value
Source: PLoS One. 2020 Jun 4;15(6):e0233959. doi: 10.1371/journal.pone.0233959 (PMC7272079; doi:10.1371/journal.pone.0233959)
Supplement: S4 Table — (DOCX) [file pone.0233959.s004.docx]

**S4 Table. Characteristics of field trials in four environments.**

| **Environment** | **Statistic** | **Yield** | **Wintering** | **Oil content** | **C18:1** | **C18:3** | **GLS** | **Flowering start** | **Flowering end** |
| --- | --- | --- | --- | --- | --- | --- | --- | --- | --- |
| B16 | mean | 19,57 | 61,01 | 44,92 | 75,80 | 6,24 | 10,95 | 117,38 | 147,21 |
|  | min | 11,58 | 32,35 | 41,76 | 63,28 | 2,25 | 5,25 | 110,00 | 135,50 |
|  | max | 26,43 | 85,50 | 47,70 | 78,43 | 8,95 | 22,43 | 122,00 | 154,00 |
| B17 | mean | 26,12 | 68,21 | 44,57 | 76,39 | 6,17 | 11,58 | 122,29 | 152,83 |
|  | min | 4,80 | 5,30 | 41,39 | 65,80 | 1,93 | 5,40 | 113,50 | 148,50 |
|  | max | 35,95 | 87,55 | 47,71 | 79,78 | 8,40 | 23,73 | 126,00 | 157,50 |
| L16 | mean | 32,36 | 74,78 | 45,88 | 77,03 | 5,40 | 9,57 | 114,76 | 146,38 |
|  | min | 3,13 | 46,90 | 42,99 | 65,78 | 2,68 | 3,03 | 109,75 | 144,25 |
|  | max | 42,20 | 94,25 | 49,13 | 81,15 | 8,23 | 18,68 | 128,00 | 154,25 |
| L17 | mean | 29,91 | 80,19 | 46,32 | 77,46 | 5,90 | 10,74 | 118,38 | 149,46 |
|  | min | 7,53 | 30,85 | 42,70 | 62,98 | 2,50 | 5,53 | 108,00 | 146,50 |
|  | max | 41,38 | 104,90 | 49,43 | 80,35 | 8,23 | 21,50 | 130,00 | 153,75 |

| Mean, mininimum and maximum values of the genotypic means for each environment; B16, Borowo 2015/2016; L16, Lagiewniki 2015/2016; B17, Borowo 2016/2017; L17, Lagiewniki 2016/2017; C18:1, oleic acid; C18:3 linolenic acid, GLS, glucosinolates. |
| --- |
